# Validity of an online 24-h recall tool (myfood24) for dietary assessment in population studies: comparison with biomarkers and standard interviews

**DOI:** 10.1186/s12916-018-1113-8

**Published:** 2018-08-09

**Authors:** Petra A. Wark, Laura J. Hardie, Gary S. Frost, Nisreen A. Alwan, Michelle Carter, Paul Elliott, Heather E. Ford, Neil Hancock, Michelle A. Morris, Umme Z. Mulla, Essra A. Noorwali, K. Petropoulou, David Murphy, Gregory D. M. Potter, Elio Riboli, Darren C. Greenwood, Janet E. Cade

**Affiliations:** 1 Centre for Innovative Research Across the Life Course (CIRAL), Faculty of Health and Life Sciences, Coventry University, Coventry, CV1 5FB UK; 2 Global eHealth Unit, Department of Primary Care and Public Health, Imperial College, Imperial College, London, SW7 2AZ UK; 3 Division of Epidemiology and Biostatistics, Leeds Institute for Cardiovascular and Metabolic Medicine, School of Medicine, University of Leeds, Leeds, LS2 9JT UK; 4 Nutrition and Dietetic Research Group, Division of Diabetes, Endocrinology and Metabolism, Faculty of Medicine, Imperial College London, London, W12 ONN UK; 5 Academic Unit of Primary Care and Population Sciences, Faculty of Medicine, University of Southampton, Southampton, SO16 6YD UK; 6 Nutritional Epidemiology Group, School of Food Science and Nutrition, University of Leeds, Leeds, LS2 9JT UK; 7 MRC-PHE Centre for Environment and Health, Department of Epidemiology and Biostatistics, School of Public Health, Faculty of Medicine, Imperial College London, London, W2 1PG UK; 8 Leeds Institute of Data Analytics, University of Leeds, Leeds, LS2 9JT UK; 9 Department of Clinical Nutrition, Faculty of Applied Medical Sciences, Umm Al-Qura University, P.O. Box 715, Makkah, 21955 Saudi Arabia; 10 NIHR Southampton Biomedical Research Centre, University of Southampton and University Hospital Southampton NHS Foundation Trust, Southampton, SO16 6YD UK

**Keywords:** Nutrition assessment, Online, Biomarkers, Validation, Nutritional epidemiology, Nutrient intake, Food, Diet, Adult

## Abstract

**Background:**

Online dietary assessment tools can reduce administrative costs and facilitate repeated dietary assessment during follow-up in large-scale studies. However, information on bias due to measurement error of such tools is limited. We developed an online 24-h recall (myfood24) and compared its performance with a traditional interviewer-administered multiple-pass 24-h recall, assessing both against biomarkers.

**Methods:**

Metabolically stable adults were recruited and completed the new online dietary recall, an interviewer-based multiple pass recall and a suite of reference measures. Longer-term dietary intake was estimated from up to 3 × 24-h recalls taken 2 weeks apart. Estimated intakes of protein, potassium and sodium were compared with urinary biomarker concentrations. Estimated total sugar intake was compared with a predictive biomarker and estimated energy intake compared with energy expenditure measured by accelerometry and calorimetry. Nutrient intakes were also compared to those derived from an interviewer-administered multiple-pass 24-h recall.

**Results:**

Biomarker samples were received from 212 participants on at least one occasion. Both self-reported dietary assessment tools led to attenuation compared to biomarkers. The online tools resulted in attenuation factors of around 0.2–0.3 and partial correlation coefficients, reflecting ranking intakes, of approximately 0.3–0.4. This was broadly similar to the more administratively burdensome interviewer-based tool. Other nutrient estimates derived from myfood24 were around 10–20% lower than those from the interviewer-based tool, with wide limits of agreement. Intraclass correlation coefficients were approximately 0.4–0.5, indicating consistent moderate agreement.

**Conclusions:**

Our findings show that, whilst results from both measures of self-reported diet are attenuated compared to biomarker measures, the myfood24 online 24-h recall is comparable to the more time-consuming and costly interviewer-based 24-h recall across a range of measures.

**Electronic supplementary material:**

The online version of this article (10.1186/s12916-018-1113-8) contains supplementary material, which is available to authorized users.

## Background

Robust assessment of the association between diet and health in population-based studies requires accurate and often repeated measurements of diet [1]. Food frequency questionnaires (FFQs), often the method of choice in large population studies, provide a convenient assessment of usual, longer-term diet. Many assumptions are made with the use of FFQs, including lists of foods likely to be consumed, portion sizes and the frequency of consumption [2]. FFQs and food recall checklists are not easy to adapt for different population groups, including ethnic minorities, due to their reliance on previously defined lists of a limited number of foods. Only a minority allow for addition of foods not listed in the pre-set food lists. The use of 24-h dietary recalls can provide more accurate intake data, with reduced measurement error, for a given day [3–5]. Use of more detailed 24-h recalls traditionally administered by trained dietitians [6] are costly to implement, with large volumes of paper-based prompts, and if not automated, they are expensive and time-consuming to code. The 24-h recalls have been prohibitively expensive for large-scale studies, especially when repeat measures are required to estimate longer-term or usual intake [1]. The capability to collect an automated, self-administered 24-h recall makes it feasible to collect multiple days of recalls to characterise usual intake in large-scale observational studies and national dietary surveys. A single recall does not capture day-to-day variation and does not allow for examination of changes in dietary patterns over time or inclusion as time-dependent covariates [7, 8]. Repeated application would reduce bias from random measurement error and allow for changes in dietary patterns. Online dietary recall systems for research have been developed and tested in the USA and some European countries. The US National Cancer Institute’s ASA24® (Automated Self-Administered 24-Hour Dietary Assessment Tool), for example, can be used to collect both 24-h dietary recalls and food records and as of August 2017 has been used by 3500 studies in the USA, Canada and Australia [9, 10]. DietDay is another online 24-h recall system being used in epidemiological research in the USA [11, 12], and a comprehensive 24-h online dietary assessment food record is being used in a large cohort study conducted in France [12, 13]. In the UK, INTAKE24 [14] and the Oxford WebQ [15] are new online tools measuring diet using a recall and questionnaire format respectively. Although most of these tools have been compared to other established dietary assessments, very few have been validated against independent dietary biomarkers [16, 17].

An automated 24-h dietary recall, such as the one we have developed and report here, has potential advantages over FFQs and interviewer-administered recalls with the opportunity for self-administration, reducing interviewer and possible coding costs. Whilst still retaining the detail of information acquired on food type and amount consumed, the number of prompts used in a paper-based system are minimised to avoid user fatigue and restricted to likely forgotten items and an overall check prior to submission. In addition, the automated system could also use a search engine of a much larger food database than, for example, the fixed food lists of FFQs or a generic food database which is commonly used in the UK for coding food diaries or recalls including 3500 foods [18]. An online searchable food database could include specific items more commonly consumed by minority groups, incorporate brand-level data and could be updated easily to reflect new products. Furthermore, such a tool could record time-varying intakes such as eating occasions, meal patterns, foods eaten in combination and portion size estimation not limited to standard portions and aided by the use of photographs. The tool we have developed is called myfood24 (Measure Your Food On One Day 24-h recall) [19], and this study aims to validate it against independent biomarkers and compare its performance with an interviewer-administered multiple-pass 24-h recall (MPR).

## Methods

### Ethics

All procedures and documents involving human subjects were reviewed and approved by the West London Research Ethics Committee (14/SC/1267) in advance of the study commencing. The study was conducted according to the guidelines of the Declaration of Helsinki, and full written informed consent was received from all participants.

### Recruitment

Participants were intended to be broadly representative of the adult general population, and to be eligible for recruitment they had to be between 18 and 65 years old and metabolically stable. This was assessed at a screening visit by confirming their weight stability defined as weight stable (gained or lost ≥3 kg weight in the past 3 months?) and their willingness to maintain current dietary and physical activity habits for the duration of the study. To complete the dietary recalls and reference measures they had to have regular high-speed Internet access and a telephone, be able to speak and read English and be willing to visit the National Institute for Health Research (NIHR)/Wellcome Trust Clinical Research Facility (CRF) at Hammersmith Hospital (Imperial College Healthcare National Health Service (NHS) Trust, London, UK). Participants were recruited through a number of sources: the North West London Primary Care Research Network (WeLReN), a multidisciplinary network of primary care professionals and practices who have expressed prior interest in participating in research projects; lists of individuals known to CRF who had previously expressed an interest in participating in research projects; posters displayed in local general practices; and through a list of local addresses obtained from the post office, with potential participants receiving a postal invitation to take part. Approximately 2000 letters were sent out.

Respondents were invited to attend a screening visit to confirm eligibility, receive a detailed explanation of the study protocol, provide informed consent and undergo a health screen. This consisted of an electrocardiogram (ECG), blood pressure, height and weight measurement, a blood sample to check routine measures of liver and kidney function and cholesterol levels and a short questionnaire. Following the screening visit, participants were randomised into the different study arms as detailed in subsequent sections. Participants were provided with £100, as compensation for their time, upon completing the study.

### Study design

**Fig. 1:**
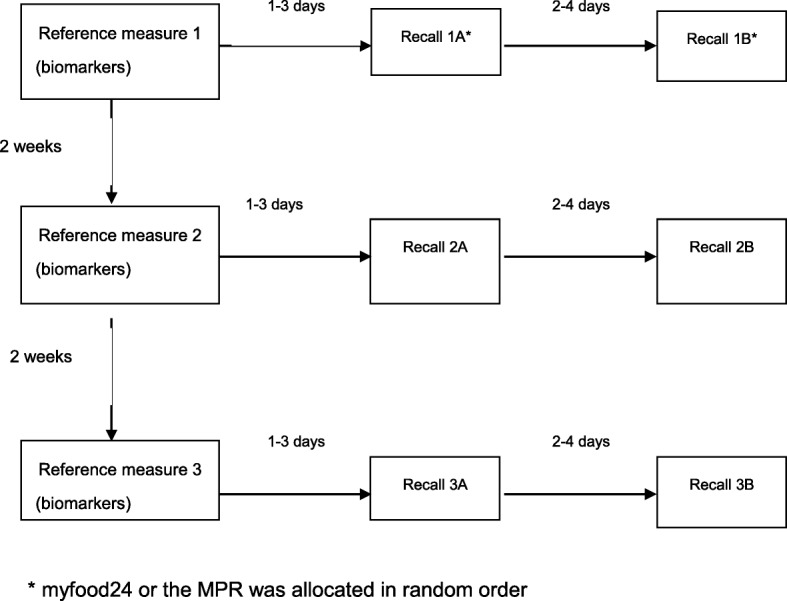
myfood24 validation study design overview. Each 24-h recall (the interviewer-based multiple-pass 24-h recall and myfood24 online tool in random order) and a suite of reference measures (biomarkers and total energy expenditure) were completed on 3 separate occasions separated by approximately 2 weeks. At each occasion, the reference measure was followed 1–3 days later by the first 24-h recall method, which was followed 2–4 days later by the second 24-h recall method

Here we report on results from two different 24-h recalls (an interviewer-based dietary MPR and the myfood24 online dietary recall). This analysis compares the standard interviewer-based recall and the online myfood24 recall against the reference measures from biomarkers. At each clinic visit, the reference measures were followed 1 to 3 days later by the first 24-h recall, which was followed approximately 2 to 4 days later by the alternative 24-h recall method. Each 24-h recall and a suite of reference measures (i.e. biomarkers and total energy expenditure) were completed on three separate occasions separated by approximately 2 weeks to approximate longer-term intake (Fig. 1). The order of the different types of 24-h recalls was allocated by simple randomisation to reduce learning effects.

### myfood24 online tool

The development of the myfood24 online 24-h dietary recall has been described in full elsewhere [19–21]. Briefly, myfood24 Version 1 was developed as an online self-administered 24-h dietary recall tool, targeting collection of automated dietary data in large-scale epidemiological studies. It was designed for speed and simplicity, requiring as few separate webpages, pop-ups and prompts as possible, but it includes an optional recipe builder, a detailed food search capability, an option to make an initial list for the first pass, prompts for commonly forgotten foods and foods often consumed together and a final review before submission. A large electronic food composition database (Version 1) was developed to reflect the variety of foods consumed in the UK. This was based on more than 3000 generic items from the UK Composition of Foods integrated dataset [18], nutrient content provided by fast food outlets and nutrient content as provided on the packaging of more than 50,000 branded food items [22] with remaining nutrients for branded items matched to the closest generic items on the basis of declared content [19]. Six thousand of the most common food items had photographic images to aid portion size recognition.

To test the online tool as an independent, stand-alone system, participants were not directly instructed in how to use myfood24 by the interviewers. However, all participants had access to a range of online help videos and frequently asked questions (https://www.youtube.com/watch?v=RI1C1Azv0Bw; https://www.youtube.com/watch?v=CpLZ_NTH_O4).

### Interviewer-administered 24-h recall

To standardise the telephone 24-h multiple-pass recall interviews, a standardised comprehensive prompt sheet based on the US Department of Agriculture (USDA) Automated Multiple-Pass Method was used by the interviewers [9]. This approach has also been applied in other myfood24 evaluation studies [20]. Nutritional intake was calculated using Dietplan 6.7 software (Forestfield Software, Horsham, UK), which is based on the McCance and Widdowson’s 6th Edition Composition of Foods UK Nutritional Dataset (UKN). A team of trained coders matched the food and drink items recorded in the recalls to UKN database codes and portion sizes using a standard operating protocol provided in a detailed supplementary document attached to this reference [23]. This protocol was developed to reduce the number of subjective decisions made by coders by providing a series of flow diagrams to guide coders in the translation of food and drink records to database codes and portion sizes to weights (grams). This has been successfully used in other studies to reduce error code rates [23]. Composite dishes were broken down into their constituent parts with the use of retailer websites to check details of ingredients.

### Urinary biomarkers

Participants were instructed to take one 80 mg 4-para-aminobenzoic acid (PABA) tablet with meals at approximately 8:00, 13:00 and 18:00 h in the 24 h preceding each study visit. They were requested to collect urine in dark containers for 24 h following the first void of the day including the first void of the next day. In addition, they recorded the timing of first and last collections, missed collections and supplement and medication use. Participants returned their 24-h urine samples to the CRF on the same day as collection ended. Urine volume was recorded before storage at − 20 °C and transportation to the Molecular Epidemiology Unit at the University of Leeds. Total urinary nitrogen was measured by the Kjeldahl method [24] with completeness of 24-h urine collection assessed by analysis of 4-para-aminobenzoic acid (PABA) concentration in the urine, using high performance liquid chromatography (HPLC) [25]. We assumed that 93% of PABA is excreted within 24 h [25] and that 81% of nitrogen is excreted within 24 h [26]. We used food-specific nitrogen-to-protein conversion ratios.

Urinary potassium and sodium concentrations were measured by the Clinical Biochemistry Department at the Leeds Teaching Hospitals NHS Trust using an ADVIA 2400 Clinical Chemistry System (Siemens AG, Munich, Germany) with ion selective electrode detection. We assumed that 80% of potassium [27] and 86% of sodium is excreted [28].

Urinary fructose and sucrose concentrations were quantified using a Sucrose/D-Glucose/D-Fructose Assay (Boehringer Mannheim/R-Biopharm AG, Darmstadt, Germany) scaled down to a microplate format. Multiplying by the total volume of urine collected over the 24 h allowed daily excretion of urinary sucrose and fructose to be estimated. This was then converted to a predicted intake of total sugars, based on a calibration equation derived from a controlled feeding study, which accounts for the age and sex of the individual [29]. As in the Observing Protein and Energy Nutrition (OPEN) study, we assumed that the relationship between urinary sucrose and fructose excretion and true intake of all sugars in our study was similar to that of an experimental sugar feeding study by Tasevska et al. [30].

### Plasma biomarkers

During participant visits to the CRF, blood samples (40 ml) were collected in lithium heparin tubes before centrifugation at 2000×g for 10 min. Plasma was collected, aliquoted and frozen at – 80 °C. Plasma concentrations of total vitamin C (dehydroascorbic and ascorbic acid), vitamin E (α-tocopherol) and β-carotene were measured by HPLC as previously described [31] in the Molecular Epidemiology Unit at the University of Leeds with detection at 270 nm for ascorbic acid, 292 nm for α-tocopherol and 452 nm for β-carotene.

### Total energy expenditure

Total energy expenditure (TEE) was estimated from combining measurements of resting energy expenditure (REE) and activity energy expenditure (AEE) and an assumed thermic effect of food. REE was measured by open-circuit indirect calorimetry (Gas Exchange Monitor; GEM Nutrition, Cheshire, UK). Following calorimeter calibration, volunteers were asked to lie in a semi-recumbent position under the canopy. Measurements were allowed to stabilise before oxygen consumption (VO_2_) and carbon dioxide production (VCO_2_) were recorded every minute for 15 min. The mean of the last 10 VO_2_ and VCO_2_ measurements was calculated and REE estimated from VO_2_ and VCO_2_ production in a given time using the equation by Weir [32]. AEE was estimated using a SenseWear three-plane accelerometer (BodyMedia Inc., Pittsburgh, PA, USA) worn on the upper arm for a period of 24 h on one of the days preceding the patient’s clinic visit. We assumed that the thermic effect of food was approximately 10% of TEE [33]. This method of estimating TEE has previously demonstrated close agreement to estimates using doubly labelled water [20, 34]. Estimated TEE served as a surrogate for total energy intake, assuming individuals were in energy balance.

### Statistical analysis

Our a priori statistical analysis plan, approved by the study team and advisors, stated that our primary comparison was both long-term and short-term agreement between myfood24 and biomarkers, compared to the agreement between the interviewer-based multiple-pass method and biomarkers. Protein was the primary dietary component since it has a well-established recovery biomarker available. All participants were included in the main analyses unless they reported missing collection of two or more voids of urine during the 24-h collection period [35] or had a greater than 5% weight change from the first clinic appointment.

Our main analysis addressed longer-term intake. The attenuation factor (the parameter measuring the ability to detect diet-disease relationships using the dietary assessment tool) and the correlation coefficient between the dietary assessment tool and estimated true long-term intake (the parameter relating to the loss of power and to attenuation of log relative risks between categories of intake) were estimated from structural equation models using the method of maximum likelihood assuming multivariate normal distributions for the data after log transformation and also assuming that any missing observations were missing at random. We assumed a similar measurement error structure to that proposed in previous validation studies [36], with self-report dietary assessments having a person-specific systematic bias as well as a systematic bias related to level of intake. We also assumed that the person-specific biases for the interviewer-based 24-h recall and myfood24 were correlated. The structural equation models included linear associations between the longer-term usual intake and both the biomarkers and self-reported intakes, as suggested by Kipnis et al. [37]. Further details of the measurement error model are provided in Additional file 1: Table S1 and section Supplementary Materials and Methods.

For recovery biomarkers and equivalent reference instruments the attenuation factors are multipliers indicating the degree to which log relative risks are attenuated because of the measurement error in the dietary assessment tool. Attenuation factors are presented for a single administration of each self-report tool. From this model the bias in both of the self-report 24-h recalls compared to the biomarkers is also estimated, based on the mean self-reported intake over the replicates for each participant minus the mean over the replicates for the biomarker or equivalent reference tool, back-transformed and expressed as a percentage. This is the equivalent to the mean difference presented in the Bland-Altman approach [38].

A sensitivity analysis was conducted including only participants with complete PABA recovery (85–110%) and adjusting the urinary nitrogen, potassium and sodium to PABA recovery of 93% where the PABA recovery was 50–85% [39]. A sensitivity analysis was also conducted excluding participants who wore their SenseWear armbands for < 23 h or > 25 h, and the main analyses were also repeated excluding 24-h recalls that were collected within 24 h of a biomarker and therefore might give an optimistic estimate of agreement longer-term.

To reflect how myfood24 may be used in practice, the attenuation factors and correlations were re-estimated based on the average of a series of 2, 4 or 7 repeat administrations of the myfood24 tool, using the same approach as that of Schatzkin et al., 2003 [40]. To assess the robustness of results to participant characteristics, analyses were repeated stratified on sex, on age and on body mass index (BMI).

For nutrients with concentration biomarkers, β-carotene, vitamin C and vitamin E, intraclass correlation coefficients (ICCs) for absolute agreement between estimated intake and the reference tool were derived from two-way mixed effects models, with the dietary assessment method as the fixed effect. We included a subject-by-method interaction to allow for different responses for the two dietary assessment tools. We allowed the variance of random coefficients to vary and measurement error variances to vary between methods and focussed on individual 24-h periods rather than averages over the three time periods [4, 41, 42].

For nutrients with no adequate biomarkers, we also estimated the ICC between estimated intakes from the two different 24-h recalls. In addition, following the approach suggested by Altman and Bland, we also presented the mean difference in estimates between the two different 24-h recalls (an estimate of relative bias), alongside estimated limits of agreement (an estimate of precision for individual measures) [38].

We applied log transformations to all our analyses. All statistical analyses were performed in Stata SE version 14.2 [43].

### Sample size

We aimed for a final sample size of 200 participants. Assuming similar parameters to those found in the OPEN study and EPIC Norfolk [37, 44], this sample size would allow the attenuation factor for protein intake to be estimated to approximately ± 0.08 and the correlation between myfood24 and true long-term intake to be estimated to approximately ± 0.1. This would also allow the mean difference between two tools to be estimated to within approximately ± 0.4 g nitrogen.

## Results

Of the 289 respondents invited to the first clinic, 84% attended, provided consent and passed the health screen. Following random allocation of order of recalls, 31 participants (13%) withdrew during the course of the study. Completed myfood24 online 24-h recalls, interviewer-based 24-h recalls and samples for biomarker analysis were provided by 212 participants on at least one occasion. There were 12 24-h collection periods amongst 11 of these participants when more than one urine sample was missed. The biomarker measurements were excluded for those occasions. However, because samples were collected on up to three occasions, no participants were excluded entirely from the study as a result.

Table 1 shows the demographic characteristics of the participants on entry to the study. The mean age of participants was 43 years, 127 (60%) were female, 155 (73%) were white and 127 (60%) were educated past age 18 years. Only 25 (12%) reported being current smokers. Mean body weight was 81 kg for male and 67 kg for female participants at the first appointment. Participants’ weights were generally stable over the course of the study, but 6 (3%) sets of energy expenditure results were excluded because of more than a 5% weight change in some participants.

**Table 1:** Demographic characteristics of participants by sex

	Men (*n* = 85)^a^	Women (*n* = 127)^a^
Mean age (years) (SD)	43 (15)	44(16)
Ethnicity		
White	63 (74%)	92 (72%)
Black	1 (1%)	9 (7%)
Asian	5 (6%)	8 (6%)
Mixed and other	15 (18%)	14 (11%)
Age left education		
16 or under	13 (15%)	11 (9%)
17 to 18	23 (27%)	36 (28%)
19+	49 (58%)	78 (61%)
Smoking status		
Non-smoker	65 (76%)	99 (78%)
Smoker	12 (14%)	13 (10%)
Mean weight (kg) (SD)	81 (13)	67 (13)
Mean body mass index (kg/m^2^) (SD)	26 (4)	25 (5)

^a^Note, where numbers in each category do not sum to the totals for the column, it is due to incomplete data for that characteristic

*SD* standard deviation

Table 2 shows the geometric mean and 95% confidence interval (CI) for protein, potassium, sodium and total sugar intakes and associated nutrient densities as assessed by the myfood24 online recall, the interviewer-based 24-h recall and the biomarkers and reference tools relating to the first clinic visit. The myfood24 estimates of intake were similar to the biomarker measurements for protein, higher for potassium and sodium and lower for total sugars and estimated total energy intake compared to reference estimates. The two types of 24-h recall gave broadly similar results, but the online myfood24 typically provided slightly lower estimates compared to the interviewer-administered tool.

**Table 2:** Geometric means and 95% confidence interval (CI) for protein, potassium, sodium and total sugar intake and density as assessed by myfood24, interviewer-based 24-h recall and biomarkers relating to the first clinic visit

	myfood24		Interviewer-based 24-h recall		Biomarker/reference tool	
	*n*	Geometric mean (95% CI)	*n*	Geometric mean (95% CI)	*n*	Geometric mean (95% CI)
Nutrient intake:						
Protein (g)	208	70.5 (66.1, 75.2)	197	81.7 (77.3, 86.4)	192	68.4 (64.1, 72.8)
Potassium (g)	208	2.7 (2.5, 2.9)	197	3.1 (3.0, 3.3)	192	2.1 (1.9, 2.3)
Sodium (g)	208	2.3 (2.1, 2.5)	197	2.4 (2.2, 2.6)	192	1.8 (1.7, 2.0)
Total sugars (g)	208	72.8 (66.4, 79.8)	197	91.0 (85.0, 97.5)	191	128.3 (115.9, 142.0)
Energy expenditure:						
Total energy expenditure (MJ)	208	7.5 (7.1, 7.9)	197	8.5 (8.1, 8.9)	185	11.0 (10.5, 11.6)
Nutrient density:^a^						
Protein (g/MJ)	208	9.5 (9.0, 9.9)	197	9.6 (9.2, 10.0)	180	6.2 (5.8, 6.7)
Potassium (g/MJ)	208	0.36 (0.35, 0.38)	197	0.37 (0.35, 0.39)	180	0.19 (0.18, 0.21)
Sodium (g/MJ)	208	0.31 (0.29, 0.33)	197	0.28 (0.27, 0.30)	180	0.16 (0.15, 0.18)
Total sugars (g/MJ)	208	9.8 (9.1, 10.5)	197	10.7 (10.1, 11.4)	179	11.6 (10.4, 12.9)

^a^Nutrient density for protein, potassium, sodium and total sugars was expressed in g/MJ of total energy intake

The n is the number of participants who had both the dietary assessment measure and the biomarker

Table 3 lists the attenuation factors for the online myfood24 and interviewer-based 24-h recalls when used to estimate long-term intake and nutrient densities. The attenuation factors, the degree to which diet-disease relationships are attenuated, were low for both self-report tools, but both were higher than those seen with FFQs [37]. The attenuation factors for the online myfood24 tool were slightly lower than those for the interviewer-based tool. The partial correlation coefficients between the self-report tools and the estimated true longer-term intake, ranging between 0.2 to 0.4, indicating the attenuation of log relative risks between categorised levels of intake as well as the loss of power introduced by measurement error, were poor for both self-report tools. For this outcome both the online and interviewer-based tools performed similarly. The mean percentage difference between the self-report tools and the biomarker measures (Table 3) reflected the extent to which the self-report tools over-estimated potassium and sodium intakes and under-estimated total sugars and total energy intake. Results for total energy intake and nutrient densities were slightly worse than those for nutrient intakes. The estimated parameters from the full measurement models are provided in Additional file 1: Table S1.

**Table 3:** Attenuation factors, correlation between dietary assessment tool and true intake and mean difference between self-report tool and reference intake for protein, potassium, sodium and total sugar intake and density as assessed by myfood24 and interviewer-based 24-h recall

	Dietary assessment tool	Attenuation factor (95% CI)	Correlation with true intake (95% CI)	Mean % difference vs reference tool (95% CI)
Nutrient intake:				
Protein (g) (*n* = 192)	myfood24	0.30 (0.21, 0.38)	0.43 (0.32, 0.53)	+1% (−4%, + 7%)
	MPR^b^	0.38 (0.29, 0.47)	0.48 (0.39, 0.58)	+ 11% (+ 6%, + 17%)
Potassium (g) (*n* = 192)	myfood24	0.31 (0.21, 0.41)	0.40 (0.28, 0.52)	+ 26% (+ 19%, + 35%)
	MPR	0.35 (0.23, 0.46)	0.38 (0.27, 0.49)	+ 48% (+ 39%, + 57%)
Sodium (g) (*n* = 192)	myfood24	0.21 (0.12, 0.30)	0.30 (0.18, 0.41)	+ 22% (+ 13%, + 32%)
	MPR	0.22 (0.11, 0.32)	0.28 (0.15, 0.40)	+ 28% (+ 18%, + 38%)
Total sugars (g) (n = 191)	myfood24	0.15 (0.06, 0.24)	0.24 (0.09, 0.38)	−45% (− 39%, − 50%)
	MPR	0.25 (0.14, 0.36)	0.31 (0.18, 0.44)	−30% (−24%, − 35%)
Energy expenditure:				
Total energy expenditure (MJ) (*n* = 185)	myfood24	0.19 (0.10, 0.29)	0.29 (0.15, 0.42)	−31% (− 27%, − 35%)
	MPR	0.32 (0.21, 0.43)	0.37 (0.25, 0.49)	−23% (− 19%, − 27%)
Nutrient density:^a^				
Protein (g/MJ) (*n* = 180)	myfood24	0.16 (0.03, 0.29)	0.17 (0.03, 0.32)	+ 48% (+ 39%, + 58%)
	MPR	0.26 (0.11, 0.40)	0.24 (0.11, 0.37)	+ 46% (+ 38%, + 55%)
Potassium (g/MJ) (*n* = 180)	myfood24	0.25 (0.09, 0.41)	0.23 (0.09, 0.37)	+ 85% (+ 72%, + 99%)
	MPR	0.38 (0.23, 0.53)	0.34 (0.22, 0.47)	+ 93% (+ 81%, + 107%)
Sodium (g/MJ) (*n* = 180)	myfood24	0.08 (−0.03, 0.19)	0.09 (−0.04, 0.21)	+ 78% (+ 63%, + 94%)
	MPR	0.11 (−0.02, 0.24)	0.11 (− 0.02, 0.24)	+ 66% (+ 52%, + 80%)
Total sugars (g/MJ) (*n* = 179)	myfood24	0.16 (0.04, 0.28)	0.21 (0.06, 0.36)	−19% (− 11%, − 26%)
	MPR	0.23 (0.09, 0.37)	0.25 (0.10, 0.39)	−8% (+ 0%, − 15%)

All dietary measures and estimates were log-transformed

^a^Nutrient density for protein, potassium, sodium and total sugars was expressed in g/MJ of total energy intake

^b^Interviewer-based multiple-pass 24-h dietary recall

Adjustment of urinary nitrogen, sodium and potassium for completeness of urine samples when PABA recovery was 50–85%, and exclusion of observations outside the range of 50–110%, led to increased derived protein (77 g vs 68 g), potassium (2.4 g vs 2.1 g) and sodium (2.1 g vs 1.8 g) intakes, which were closer to the self-reported intakes. This did not substantially influence the estimates of the attenuation factors (0.27 vs 0.30 for protein, 0.29 vs 0.31 for potassium and 0.19 vs 0.21 for sodium). However, the correlation between self-report intakes and true intake was somewhat improved, albeit with wider confidence intervals (0.50 vs 0.43 for protein, 0.48 vs 0.40 for potassium and 0.37 vs 0.30 for sodium).

Attenuation factors were almost identical when participants who did not wear their armbands for 24 h were excluded (data not shown). When intakes estimated within 24 h of a biomarker collection were excluded, attenuation factors were marginally lower for protein, potassium and sodium, and essentially unchanged for total sugars and total energy intake, but all with wider confidence intervals (data not shown).

Using the average of a series of 2, 4 or 7 repeat administrations, attenuation was reduced and correlations improved with repeat administration of the myfood24 tool (Additional file 1: Table S2).

Attenuation factors from models stratified by BMI showed that BMI may modify attenuation factors and deviations from true values when using myfood24 and the traditional 24 h recall (Table 4), with somewhat lower attenuation for participants with BMI < 25 kg/m^2^ for measures of protein and potassium. Other models stratifying by sex and age found attenuation of total energy intake and nutrient densities slightly better for males compared to females, and less attenuation for most nutrients with younger age (Additional file 1: Tables S3 and S4).

**Table 4:** Attenuation factors and correlation between dietary assessment tool and true intake for protein, potassium, sodium and total sugar intake and density as assessed by myfood24 and interviewer-based 24-h recall by body mass index

	Dietary assessment tool	Body mass index < 25 kg/m^2^ (*n* = 99)		Body mass index 25+ kg/m^2^ (*n* = 99)	
		Attenuation factor (95% CI)	Correlation with true intake (95% CI)	Attenuation factor (95% CI)	Correlation with true intake (95% CI)
Nutrient intake:					
Protein (g)	myfood24	0.32 (0.20, 0.45)	0.45 (0.30, 0.60)	0.28 (0.16, 0.40)	0.42 (0.26, 0.57)
	MPR^b^	0.37 (0.24, 0.50)	0.44 (0.32, 0.57)	0.33 (0.21, 0.45)	0.47 (0.33, 0.61)
Potassium (g)	myfood24	0.43 (0.28, 0.59)	0.51 (0.36, 0.66)	0.18 (0.04, 0.32)	0.40 (0.26, 0.54)
	MPR	0.40 (0.23, 0.57)	0.26 (0.06, 0.45)	0.27 (0.12, 0.41)	0.34 (0.18, 0.51)
Sodium (g)	myfood24	0.17 (0.04, 0.30)	0.24 (0.07, 0.42)	0.24 (0.12, 0.35)	0.34 (0.19, 0.49)
	MPR	0.17 (0.05, 0.29)	0.24 (0.08, 0.41)	0.15 (0.04, 0.26)	0.22 (0.07, 0.37)
Total sugars (g)	myfood24	0.14 (0.02, 0.26)	0.25 (0.04, 0.47)	0.14 (−0.01, 0.28)	0.19 (− 0.01, 0.39)
	MPR	0.14 (0.02, 0.26)	0.22 (0.05, 0.40)	0.24 (0.10, 0.37)	0.30 (0.14, 0.45)
Energy expenditure:					
Total energy expenditure (MJ)	myfood24	0.16 (0.04, 0.29)	0.28 (0.07, 0.48)	0.20 (0.06, 0.33)	0.27 (0.09, 0.45)
	MPR	0.27 (0.14, 0.39)	0.39 (0.23, 0.56)	0.23 (0.08, 0.39)	0.27 (0.11, 0.43)
Nutrient density:^a^					
Protein (g/MJ)	myfood24	0.29 (0.11, 0.48)	0.32 (0.14, 0.51)	−0.06 (− 0.25, 0.13)	−0.06 (− 0.27, 0.14)
	MPR	0.34 (0.17, 0.51)	0.34 (0.20, 0.49)	0.04 (−0.13, 0.20)	0.04 (−0.14, 0.23)
Potassium (g/MJ)	myfood24	0.35 (0.11, 0.59)	0.29 (0.10, 0.48)	0.15 (−0.07, 0.36)	0.15 (−0.07, 0.37)
	MPR	0.62 (0.37, 0.87)	0.47 (0.31, 0.62)	0.24 (0.01, 0.46)	0.24 (0.02, 0.46)
Sodium (g/MJ)	myfood24	0.05 (−0.13, 0.22)	0.05 (−0.13, 0.23)	0.14 (− 0.02, 0.29)	0.16 (− 0.01, 0.33)
	MPR	0.12 (−0.04, 0.28)	0.13 (− 0.04, 0.31)	0.07 (− 0.06, 0.21)	0.09 (− 0.07, 0.25)
Total sugars (g/MJ)	myfood24	0.07 (− 0.08, 0.22)	0.12 (− 0.15, 0.38)	0.24 (0.05, 0.43)	0.27 (0.07, 0.47)
	MPR	0.08 (−0.06, 0.22)	0.14 (−0.09, 0.37)	0.27 (0.09, 0.44)	0.28 (0.11, 0.44)

All dietary measures and estimates were log-transformed

^a^Nutrient density for protein, potassium, sodium and total sugars was expressed in g/MJ of total energy intake

^b^Interviewer-based multiple-pass 24-h dietary recall

The intraclass correlation (95% CI) between plasma antioxidant concentrations and estimated intake from myfood24 were 0.56 (0.52, 0.60), 0.53 (0.50, 0.57) and 0.55 (0.50, 0.59) for β-carotene, vitamin C and vitamin E respectively (Table 5). These correlations were very similar to those obtained using the interviewer-based 24-h recall.

**Table 5:** Geometric mean biomarker concentration, estimated intake at first dietary recall by myfood24 and interviewer-based tool and intraclass correlation coefficients between biomarker and each tool assessed over three time points

	Biomarker *n* = 202	myfood24 *n* = 208		Interviewer-based 24-h recall *n* = 197	
	Geometric mean (95% CI)	Geometric mean (95% CI)	Intraclass correlation with biomarker	Geometric mean (95% CI)	Intraclass correlation with biomarker
Nutrient intake:					
β-carotene	0.59 μM (0.52, 0.67)	0.65 mg (0.51, 0.83)	0.56 (0.52, 0.60)	1.53 mg (1.20, 1.95)	0.52 (0.48, 0.56)
Vitamin C	60 μM (57, 64)	59 mg (51, 69)	0.53 (0.50, 0.57)	75 mg (66, 85)	0.53 (0.49, 0.56)
Vitamin E	37 μM (35, 40)	1.6 mg (1.3, 1.9)	0.55 (0.50, 0.59)	2.3 mg (2.0, 2.8)	0.53 (0.49, 0.57)

Table 6 shows the geometric mean intake and 95% CI for each nutrient estimated by the myfood24 online tool and the interviewer-administered tool at the time of first recall. It also presents the percent difference between the two methods with 95% CI, the Bland-Altman limits of agreement between the two methods and the intraclass correlation between the two methods, with 95% CI. The myfood24 estimates of nutrient intake were around 10–20% lower compared to the interviewer-based estimates. The ICCs comparing myfood24 and interviewer-based estimates were generally in the range 0.4–0.5, indicating moderate agreement between the two methods.

**Table 6:** Geometric mean at first dietary recall, percent difference in means over all recalls, limits of agreement and intraclass correlation coefficients for nutrient intake estimated from myfood24 and interviewer-based 24-h recalls

	myfood24 *n* = 208	Interviewer-based 24-h recall *n* = 197			
	Geometric mean (95% CI)	Geometric mean (95% CI)	% Difference in means^a^	% Limits of agreement^a^	Intraclass correlation
Nutrient intake:					
Total energy intake (MJ)	7.5 (7.1, 7.9)	8.5 (8.1, 8.9)	−10% (− 14%, − 6%)	− 63% to + 118%	0.51 (0.48, 0.54)
Fat (g)	68 (64, 73)	82 (77, 88)	−14% (−18%, − 9%)	− 74% to + 182%	0.42 (0.40, 0.45)
Saturated fatty acids per 100 g food (g)	23 (21, 25)	28 (25, 30)	−13% (− 19%, − 8%)	− 79% to + 263%	0.40 (0.38, 0.43)
Monounsaturated fatty acids per 100 g food (g)	24 (22, 26)	28 (26, 30)	− 14% (− 19%, − 8%)	− 78% to + 238%	0.40 (0.38, 0.43)
Polyunsaturated fatty acids per 100 g food (g)	12 (11, 13)	14 (13, 15)	−11% (−17%, −5%)	− 80% to + 303%	0.39 (0.36, 0.41)
Protein (g)	72 (68, 78)	78 (73, 82)	−9% (− 14%, − 5%)	− 68% to + 158%	0.45 (0.42, 0.48)
Carbohydrate (g)	198 (186, 211)	224 (213, 236)	−12% (−17%, − 7%)	− 70% to + 162%	0.54 (0.51, 0.56)
Starch (g)	107 (99, 116)	117 (110, 126)	− 9% (− 15%, − 3%)	− 79% to + 263%	0.44 (0.41, 0.46)
Total sugars (g)	73 (67, 80)	92 (86, 98)	−20% (− 25%, − 15%)	−80% to + 216%	0.46 (0.43, 0.49)
Alcohol (g)	1.3 (1.0, 1.6)	1.5 (1.2, 2.0)	−9% (− 23%, + 7%)	− 98% to + 4177%	0.38 (0.35, 0.40)
Englyst fibre (g)	14 (13, 15)	15 (14, 16)	−14% (− 19%, − 9%)	−75% to + 199%	0.43 (0.41, 0.46)
Cholesterol (g)	0.17 (0.15, 0.20)	0.21 (0.18, 0.24)	−12% (− 20%, − 3%)	− 91% to + 793%	0.37 (0.35, 0.39)
Sodium (g)	2.3 (2.1, 2.5)	2.4 (2.2, 2.6)	−4% (− 10%, + 2%)	− 76% to + 283%	0.44 (0.41, 0.47)
Potassium (g)	2.7 (2.5, 2.9)	3.1 (3.0, 3.3)	− 14% (−18%, − 10%)	− 69% to + 137%	0.48 (0.45, 0.51)
Calcium (g)	0.68 (0.63, 0.74)	0.83 (0.77, 0.89)	−7% (− 22%, − 12%)	−79% to + 223%	0.46 (0.43, 0.49)
Phosphorous (g)	1.2 (1.1, 1.3)	1.4 (1.3, 1.4)	−12% (− 16%, −8%)	−68% to + 143%	0.51 (0.48, 0.53)
Iron (mg)	11.3 (10.6, 12.0)	12.3 (11.6, 13.0)	−10% (−14%, − 5%)	− 70% to + 170%	0.46 (0.43, 0.49)
Copper (mg)	1.8 (1.7, 1.9)	1.8 (1.7, 1.8)	−2% (−6%, + 2%)	− 63% to + 162%	0.42 (0.39, 0.45)
Zinc (mg)	8.6 (8.0, 9.2)	10.0 (9.4, 10.6)	−10% (−14%, −4%)	−72% to + 197%	0.46 (0.43, 0.48)
Selenium (μg)	38 (33, 42)	44 (40, 48)	−13% (−20%, −6%)	− 86% to + 445%	0.43 (0.40, 0.45)
Iodine (μg)	85 (75, 96)	128 (116, 140)	−27% (− 33%, −9%)	−91% to + 509%	0.44 (0.42, 0.47)
Retinol (μg)	143 (114, 179)	218 (181, 262)	−27% (− 36%, − 15%)	− 98% to + 2188%	0.37 (0.35, 0.40)
Vitamin D (μg)	2.1 (1.9, 2.4)	2.2 (1.9, 2.5)	−3% (− 13%, + 7%)	−91% to + 908%	0.37 (0.35, 0.39)
Vitamin E (mg)	7.7 (6.9, 8.5)	9.7 (8.9, 10.5)	−19% (− 25%, −12%)	−86% to + 381%	0.43 (0.40, 0.46)
Thiamin (mg)	1.9 (1.8, 2.0)	1.9 (1.9, 2.0)	−6% (−9%, −2%)	− 61% to + 129%	0.46 (0.43, 0.49)
Niacin (mg)	18.7 (17.4, 20.2)	20.0 (18.5, 21.5)	−5% (−10%, + 1%)	−75% to + 265%	0.41 (0.38, 0.43)
Vitamin B6 (mg)	2.2 (2.1, 2.3)	2.5 (2.4, 2.7)	−10% (−14%, −7%)	− 65% to 127%	0.42 (0.39, 0.45)
Vitamin B12 (μg)	3.4 (3.1, 3.9)	5.2 (4.8, 5.8)	−27% (− 33%, − 21%)	−90% to + 422%	0.40 (0.38, 0.43)
Folate (mg)	.22 (.20, .24)	.25 (.24, .27)	−14% (− 21%, −11%)	−78% to + 215%	0.45 (0.42, 0.48)
Pantothenic acid (mg)	4.6 (4.3, 4.9)	5.9 (5.5, 6.2)	−20% (− 24%, − 16%)	−74% to + 150%	0.43 (0.41, 0.46)
Biotin (μg)	30 (28, 33)	37 (35, 40)	−19% (−25%, −13%)	−81% to + 240%	0.45 (0.42, 0.48)
Vitamin C (mg)	59 (51, 69)	76 (66, 86)	−28% (− 36%, −20%)	−94% to + 765%	0.45 (0.42, 0.47)

^a^The difference in means and limits of agreement relate to the ratio of geometric means because of log transformation and are presented as % differences

## Discussion

Our findings show that the myfood24 online 24-h recall is comparable to the more time-consuming and costly interviewer-based 24-h recall across a range of dietary measures. This is in line with previous reviews of online and computer-based dietary assessment tools [10, 45, 46]. Whilst both the online and interviewer-based 24 h recall tools suffer from the same problems of measurement error and correlated person-specific biases [47] to which all self-report tools are prone [39], they both perform broadly as well as other 24-h recalls in the USA, and most importantly, substantially better than widely used FFQs [3, 5, 37].

Our statistical approach to method validation was strong, although we did not set an a priori level of validity, which is not common practice in validation of dietary assessment tools, as shown by 78 validated tools included on the Nutritools website [48]. Rather than use Pearson’s correlation, which tends to give a falsely optimistic view of a dietary assessment tool and does not provide a measure of agreement [38], we have used measures of agreement that estimate the extent to which the diet-disease association to be estimated in a large-scale study would be attenuated if the tool were being used, either to provide a continuous estimate of nutrient intake, or ranking intake in categories. The correlation coefficients reported in our paper are akin to intraclass correlations measuring agreement. In this way, we demonstrate the utility of the tool in practice and allow comparison with other tools assessed in the same way. As such, the myfood24 tool is better than FFQs and performs similarly to dietary MPRs assessed in the USA where similar approaches to validation have been used [37].

The mean percentage differences showed that both self-report tools under-estimated intakes of total sugars and energy and over-estimated intakes of potassium and sodium in comparison to the biomarkers. In this regard, use of the myfood24 tool is no better on a population level than an interviewer-based 24 h recall, which is prone to the same problems of under-reporting and over-reporting. This is also true of other self-report tools used in national surveys, such as the 4-day food diary currently used in the UK National Diet and Nutrition Survey (NDNS), where total energy intake is mis-reported by 46% in adults aged 16–64 years [49]. This could be as a result of systematic under-estimation or over-estimation using self-report measurement tools. Reporting error, daily variation in diet and limitations of food composition tables can all affect results. For example, although potassium intake may be captured accurately using self-report methods, the ability to assess sodium intake is more controversial as a result of addition of salt to foods in manufacture or at the table [5]. Biomarkers as a gold standard may also have limitations; for example, predictive biomarkers such as urinary sucrose and fructose may vary as a result of between-person differences in sucrose and fructose absorption, uptake by tissues or reabsorption in the kidneys [50].

The extent of under-reporting or over-reporting of self-reported dietary assessment tools compared to objective biomarkers is rarely reported as transparently as we have done here. Although the attenuation associated with using nutrient intakes derived from myfood24 is in the order of 0.2–0.3, and the attenuation associated with categorised intakes from myfood24 in the order of 0.3–0.4, the equivalent attenuation bias results from some FFQs have previously shown to be much worse, with attenuation factors almost half these figures. For example, the OPEN Study, including three different and well-used FFQs, showed attenuation factors for protein compared to a biomarker of 0.16 for men and 0.14 for women [37]. A more recently developed FFQ in the Netherlands fared somewhat better, with an adjusted attenuation factor of 0.28 for protein intake against the biomarker [51]. The favourable properties of the myfood24 tool do not simply reflect the close administration of the tool relative to biomarker sampling for a proportion of the recalls, as attenuation factors were similar when any intakes estimated within 24 h of a biomarker collection were excluded.

The agreement between plasma antioxidants and estimated intakes was good in our setting, as indicated by ICCs between 0.5 and 0.6. Between interviewer-administered and Internet-administered 24-h recalls coefficients were generally in the range of 0.4–0.5, indicating moderate agreement, but there was a tendency for the online tool to be lower for most nutrients by around 10–20%. This relative bias may imply that myfood24 under-estimates, or the traditional interviewer-based multiple pass recall over-estimates, or that both tools are biased compared to the truth. For estimated intakes for individuals, this relative bias may be important, but it may be less important when diet is categorised and ranked, as is common in reporting large-scale epidemiological studies. As with all comparisons of dietary assessments conducted over different days, the limits of agreement were wide, reflecting the wide day-to-day variation in diets.

Sensitivity analyses found potentially lower attenuation for participants with BMI < 25 kg/m^2^ for measures of protein and potassium, highlighting the need to take account of participant characteristics when measuring diet.

Some previous comparisons between interviewer-administered 24-h recalls over the telephone and self-administered 24-h recalls over the Internet compared dietary intake on the same day using the same computerised interface for data entry [12, 13]. Both the same 24-h period and the same data entry interface lead to much closer agreement between the self-administered and interviewer-administered approaches, but either could lead to potentially substantial learning effects, as participants may simply repeat what was recalled using the previous tool. The same data entry interface also eliminates differences in estimated portion size options between the two approaches, exaggerating the apparent agreement. Finally, using the same 24-h period means that the validation relates to short-term use, rather than longer-term average intake, where agreement would be expected to be lower anyway. By contrast, our methodology directly assesses agreement between the two dietary assessment tools, rather than correlation which would exaggerate similarities between the tools [28], and it does not use the same day for each tool. Our focus is on agreement in estimated longer-term intake, by spreading different dietary assessments over a number of weeks, each recorded at different time points [52].

For large-scale prospective studies and health surveys, FFQs are the common choice due to interviewer-based 24-h recalls being prohibitively expensive to administer in person or over the telephone by trained researchers, and time-consuming to identify food items and analyse for nutrient content, despite the 24-h recall capturing intake with less bias in validation studies [3]. The myfood24 tool has a substantially better measurement error profile than many FFQs and would lead to less attenuation of diet-disease association estimates and greater power to detect associations as statistically significant, particularly given the lower attenuation following repeat administrations. Moreover, for the online myfood24 tool there would be negligible additional financial and staff costs associated with increasing the number of 24-h recalls that each individual provides, contrasting with costly interviewer-based 24-h recalls. This allows greater precision of estimated longer-term intake, for intake to be assessed across different seasons, for estimated intake to be updated frequently throughout follow-up, improved capture of episodically consumed items and for intra-person variability to be estimated as well as inter-person variability [1]. We believe that these strengths make online 24-h recall such as myfood24 the tool of choice for future large-scale studies, either alone or in combination with FFQs, potentially addressing some of the current criticism of self-reported dietary data.

We have compared the myfood24 tool with objective biomarker measures that are not prone to person-specific bias that might be correlated with the self-report tool being assessed. This provides a better evaluation of the tool than a comparison with another self-report 24-h recall alone, which would be prone to similar measurement errors and might be equally poor. The use of objective reference measures such as biomarkers of intake and energy expenditure is therefore a major strength of our validation study.

One weakness of our study was not being able to use the gold standard measure for energy of doubly labelled water because of the prohibitive expense [3]. However, the activity monitor equipment we used provides an alternative measure of TEE that is also objective and therefore meets the same purpose. However, this might explain why agreement between biomarker measures for nutrient densities was not as high as that for the absolute nutrients. Another weakness common to all validation studies using biomarkers is that most nutrients do not have adequate recovery biomarkers with which to validate estimated intake [53]. Whilst other objective measures exist that could be used for such nutrients, such as larder inventories and itemised till receipts, these have their own weaknesses such as being measured at a household level and not allowing for food waste [54].

One further potential challenge of this online approach is that Internet-based tools may be more acceptable to younger than to older people, and may be more accessible to individuals who are more educated or have greater income. However, our study covered a wide age range, including anyone between 18 and 65 years old, with a mean age in the middle of this range. Our participants were motivated and reimbursed for participation. Use of an Internet tool is no substitute for good study design and consideration of approaches to maximise participation rates. In developing the tool we assessed the acceptability of the tool in different age groups [19–21] and in pregnancy [55], with system usability scores being ‘good’. When we stratified our results by age group, by sex and by BMI, the myfood24 tool appeared to offer less attenuation with younger participants who were not obese. However, the same was seen for the interviewer-based tool as well. Furthermore, the unique use of brand-specific nutrient information [19, 22] means that in principle the tool is more able to match the different diets found across different demographic groups around the country.

These encouraging results provide a platform to develop country-specific versions of the tool, incorporating local foods, with estimated nutrient intakes based on food composition tables local to that country.

## Conclusions

To conclude, whilst all self-report tools are prone to substantial measurement error and associated bias, the attenuation and bias from the online myfood24 tool are substantially better than those of many alternative FFQs. The estimated attenuation factors for the myfood24 tool are similar to those of the more resource-consuming expert interviewer-administered MPR. It is therefore more likely to be of use in large-scale population surveys, prospective cohorts and trials to give more valid estimates of diet-disease associations than an FFQ and may be able to better measure the effect of dietary exposures on health and disease outcomes.

## Additional file

Additional file 1: **Table S1.** Measurement error structure for protein, potassium, sodium and total sugar intake and density as assessed by myfood24 and interviewer-based 24-h recall. **Table S2.** Attenuation factors and correlation between dietary assessment tool and true intake for protein, potassium, sodium and total sugar intake as assessed by myfood24 for different numbers of repeat administrations of the tool. **Table S3.** Attenuation factors and correlation between dietary assessment tool and true intake for protein, potassium, sodium and total sugar intake and density as assessed by myfood24 and interviewer-based 24-h recall by sex. **Table S4.** Attenuation factors and correlation between dietary assessment tool and true intake for protein, potassium, sodium and total sugar intake and density as assessed by myfood24 and interviewer-based 24-h recall by age group. (DOCX 27 kb)

## Abbreviations

BMI: Body mass index
FFQ: Food frequency questionnaire
ICC: Intraclass correlation coefficient
