# Supplementary material for: Validity of an online 24-h recall tool (myfood24) for dietary assessment in population studies: comparison with biomarkers and standard interviews
Source: BMC Med. 2018 Aug 9;16:136. doi: 10.1186/s12916-018-1113-8 (PMC6083628; doi:10.1186/s12916-018-1113-8)
Supplement: Supplementary file 1 — Table S1. Measurement error structure for protein, potassium, sodium and total sugar intake and density as assessed by myfood24 and interviewer-based 24-h recall. Table S2. Attenuation factors and correlation between dietary assessment tool and true intake for protein, potassium, sodium and total sugar intake as assessed by myfood24 for different numbers of repeat administrations of the tool. Table S3. Attenuation factors and correlation between dietary assessment tool and true intake for protein, potassium, sodium and total sugar intake and density as assessed by myfood24 and interviewer-based 24-h recall by sex. Table S4. Attenuation factors and correlation between dietary assessment tool and true intake for protein, potassium, sodium and total sugar intake and density as assessed by myfood24 and interviewer-based 24-h recall by age group. (DOCX 27 kb) [file 12916_2018_1113_MOESM1_ESM.docx]

**Additional file**

Supplementary Materials and Methods

*Measurement error model*

Our measurement error model for total energy intake, protein, potassium and sodium intakes and densities, follows that proposed by Kipnis *et al*. [refs] for myfood24 estimate *Q_ij_*, interviewer-based 24-hour recall *F_ij_* and reference measure *M_ij_* on person *i* at occasion *j*:

*Q_ij_* = μ*_Qj_* + β*_Q_*_0_ + β*_Q_*_1_*T_i_* + *r_i_* + ε*_ij_*

*F_ij_* = μ*_Fj_* + β*_F_*_0_ + β*_F_*_1_*T_i_* + *s_i_* + *u_ij_*

*M_ij_* = μ*_Mj_* + *T_i_* + *v_ij_*

where *T_i_* is the true intake for individual *i*, μ*_Qj_* and μ*_Fj_* represent a possible drift over the time period between measures, to improve model fit; β*_Q_*_0_, β*_Q_*_1_, β*_F_*_0_ and β*_F_*_1_ are biases where β*_Q_*_0_ and β*_F_*_0_ are additive components associated with the instruments used, and β*_Q_*_1_ and β*_F_*_1_ are multiplicative components; *r_i_* and *s_i_* model the person-specific bias for each tool. We allow these person-specific biases to be correlated, with correlation ρ(r,s)≠0, because the same mechanisms may be influencing both *r_i_* and *s_i_*. We assume within-person errors ε*_ij_* and *u_ij_* are independent of each other and follow normal distributions with zero mean and variances σ_ε_^2^ and σ*_u_*^2^ respectively. We assume that there is no person-specific bias associated with the reference tool *M_ij_* and that the within-person error *v_ij_* follows a normal distribution with zero mean, variance σ*_v_*^2^, and is independent of the true intake and other error components. For analyses assessing estimated intake from myfood24 based on the average of k serial measurements, variances σ_ε_^2^ is replaced by σ_ε_^2^/k.

Table S1. Measurement error structure for protein, potassium, sodium and total sugar intake and density as assessed by myfood24 and interviewer-based 24-hour recall.^‡^

|  | Variance of true intake (95% CI)  (σ*_T_*^2^) | Dietary assessment tool | Slope in regression of reported intake on true intake (95% CI)  (β*_Q_*_1 or_ β*_F_*_1_) | Variance of person-specific bias (95% CI)  (σ*_r_*^2^ _or_ σ*_s_*^2^) | Correlation between person-specific biases (95% CI)  (ρ_(r,s)_) | Variance of within-person error (95% CI)  (σ_ε_^2^ _or_ σ*_u_*^2^) |
| --- | --- | --- | --- | --- | --- | --- |
| *Nutrient intake*: |  |  |  |  |  |  |
| Protein (g) | 0.11 (0.08, 0.14) | myfood24  MPR^†^ | 0.62 (0.44, 0.79)  0.61 (0.47, 0.76) | 0.05 (0.03, 0.08)  0.02 (0.01, 0.05) | 0.57 (0.26, 0.89) | 0.13 (0.11, 0.15)  0.11 (0.09, 0.13) |
| Potassium (g) | 0.13 (0.09, 0.17) | myfood24  MPR | 0.51 (0.33, 0.68)  0.42 (0.28, 0.56) | 0.07 (0.05, 0.10)  0.03 (0.02, 0.05) | 0.59 (0.35, 0.82) | 0.11 (0.09, 0.13)  0.10 (0.09, 0.11) |
| Sodium (g) | 0.17 (0.12, 0.23) | myfood24  MPR | 0.42 (0.24, 0.60)  0.35 (0.18, 0.52) | 0.06 (0.03, 0.10)  0.07 (0.05, 0.11) | 0.60 (0.30, 0.90) | 0.25 (0.21, 0.29)  0.17 (0.15, 0.20) |
| Total sugars (g) | 0.17 (0.12, 0.24) | myfood24  MPR | 0.38 (0.14, 0.63)  0.38 (0.21, 0.56) | 0.18 (0.13, 0.24)  0.07 (0.04, 0.10) | 0.79 (0.60, 0.97) | 0.24 (0.20, 0.28)  0.17 (0.14, 0.19) |
| *Energy expenditure:* |  |  |  |  |  |  |
| Total energy expenditure (MJ) | 0.08 (0.06, 0.10) | myfood24  MPR | 0.42 (0.21, 0.64)  0.44 (0.28, 0.60) | 0.07 (0.05, 0.10)  0.02 (0.02, 0.04) | 0.60 (0.39, 0.81) | 0.08 (0.07, 0.10)  0.06 (0.06, 0.07) |
|  |  |  |  |  |  |  |
| *Nutrient density**: |  |  |  |  |  |  |
| Protein (g / MJ) | 0.10 (0.07, 0.14) | myfood24  MPR | 0.19 (0.03, 0.35)  0.23 (0.09, 0.36) | 0.03 (0.02, 0.05)  0.02 (0.02, 0.04) | 0.80 (0.58, 1.02) | 0.08 (0.07, 0.09)  0.06 (0.05, 0.07) |
| Potassium (g / MJ) | 0.14 (0.10, 0.20) | myfood24  MPR | 0.21 (0.08, 0.35)  0.31 (0.18, 0.44) | 0.05 (0.03, 0.06)  0.03 (0.02, 0.04) | 0.92 (0.73, 1.12) | 0.07 (0.06, 0.08)  0.07 (0.06, 0.09) |
| Sodium (g / MJ) | 0.18 (0.12, 0.25) | myfood24  MPR | 0.10 (-0.04, 0.23)  0.12 (-0.02, 0.25) | 0.02 (0.00, 0.06)  0.03 (0.02, 0.06) | 0.60 (0.01, 1.19) | 0.19 (0.16, 0.22)  0.15 (0.13, 0.18) |
| Total sugars (g / MJ) | 0.16 (0.10, 0.25) | myfood24  MPR | 0.27 (0.06, 0.49)  0.27 (0.09, 0.44) | 0.09 (0.06, 0.14)  0.06 (0.04, 0.08) | 1.00 (0.83, 1.17) | 0.17 (0.15, 0.20)  0.12 (0.11, 0.14) |

* nutrient density for protein, potassium, sodium and total sugars was expressed in grams per MJ of total energy intake

^†^ Interviewer-based multiple-pass 24-hour dietary recall

^‡^ All dietary measures and estimates were log-transformed.

Table S2. Attenuation factors and correlation between dietary assessment tool and true intake for protein, potassium, sodium and total sugar intake as assessed by myfood24 for different numbers of repeat administrations of the tool.^†^

|  | Number of repeat administrations | Attenuation factor  (95% CI) | Correlation with true intake  (95% CI) |
| --- | --- | --- | --- |
| Protein (g) | 1  2  4  7 | 0.30 (0.21, 0.38)  0.42 (0.30, 0.53)  0.52 (0.38, 0.67)  0.59 (0.42, 0.75) | 0.43 (0.32, 0.53)  0.51 (0.39, 0.63)  0.57 (0.44, 0.70)  0.60 (0.46, 0.74) |
| Potassium (g) | 1  2  4  7 | 0.31 (0.21, 0.41)  0.42 (0.28, 0.56)  0.51 (0.34, 0.67)  0.56 (0.37, 0.74) | 0.40 (0.28, 0.52)  0.46 (0.32, 0.60)  0.51 (0.36, 0.65)  0.53 (0.38, 0.68) |
| Sodium (g) | 1  2  4  7 | 0.21 (0.12, 0.30)  0.34 (0.20, 0.47)  0.48 (0.28, 0.67)  0.58 (0.34, 0.83) | 0.30 (0.18, 0.41)  0.38 (0.23, 0.52)  0.45 (0.28, 0.61)  0.49 (0.31, 0.68) |
| Total sugars (g) | 1  2  4  7 | 0.15 (0.06, 0.24)  0.20 (0.08, 0.33)  0.25 (0.09, 0.40)  0.27 (0.10, 0.44) | 0.24 (0.09, 0.38)  0.28 (0.11, 0.45)  0.31 (0.12, 0.49)  0.32 (0.13, 0.52) |
| Total energy expenditure (MJ) | 1  2  4  7 | 0.19 (0.10, 0.29)  0.26 (0.13, 0.38)  0.31 (0.16, 0.46)  0.34 (0.17, 0.50) | 0.29 (0.15, 0.42)  0.33 (0.18, 0.48)  0.36 (0.19, 0.53)  0.38 (0.20, 0.55) |

* nutrient density for protein, potassium, sodium and total sugars was expressed in grams per MJ of total energy intake

^†^ All dietary measures and estimates were log-transformed.

Table S3. Attenuation factors and correlation between dietary assessment tool and true intake for protein, potassium, sodium and total sugar intake and density as assessed by myfood24 and interviewer-based 24-hour recall by sex.^‡^

|  | Dietary assessment tool | Men (n=85) | |  | Women (n=127) | |
| --- | --- | --- | --- | --- | --- | --- |
|  |  | Attenuation factor  (95% CI) | Correlation with true intake  (95% CI) |  | Attenuation factor  (95% CI) | Correlation with true intake  (95% CI) |
| *Nutrient intake*: |  |  |  |  |  |  |
| Protein (g) | myfood24  MPR^†^ | 0.23 (0.13, 0.34)  0.30 (0.19, 0.41) | 0.38 (0.26, 0.51)  0.45 (0.34, 0.55) |  | 0.20 (0.10, 0.30)  0.21 (0.11, 0.30) | 0.36 (0.20, 0.52)  0.35 (0.20, 0.49) |
| Potassium (g) | myfood24  MPR | 0.26 (0.13, 0.40)  0.36 (0.22, 0.51) | 0.37 (0.21, 0.53)  0.43 (0.29, 0.56) |  | 0.31 (0.17, 0.44)  0.28 (0.14, 0.41) | 0.40 (0.24, 0.56)  0.34 (0.18, 0.49) |
| Sodium (g) | myfood24  MPR | 0.18 (0.09, 0.28)  0.18 (0.09, 0.27) | 0.35 (0.22, 0.48)  0.34 (0.22, 0.46) |  | 0.17 (0.06, 0.29)  0.11 (0.01, 0.22) | 0.25 (0.09, 0.41)  0.16 (0.01, 0.32) |
| Total sugars (g) | myfood24  MPR | 0.14 (0.02, 0.26)  0.24 (0.12, 0.36) | 0.25 (0.05, 0.44)  0.37 (0.23, 0.52) |  | 0.13 (0.01, 0.25)  0.14 (0.03, 0.25) | 0.22 (0.02, 0.41)  0.20 (0.04, 0.36) |
| *Energy expenditure:* |  |  |  |  |  |  |
| Total energy expenditure (MJ) | myfood24  MPR | 0.10 (0.03, 0.18)  0.16 (0.05, 0.26) | 0.26 (0.14, 0.39)  0.33 (0.21, 0.46) |  | 0.04 (-0.08, 0.16)  0.08 (-0.05, 0.20) | 0.07 (-0.13, 0.27)  0.10 (-0.06, 0.27) |
|  |  |  |  |  |  |  |
| *Nutrient density**: |  |  |  |  |  |  |
| Protein (g / MJ) | myfood24  MPR | 0.20 (0.00, 0.41)  0.20 (0.02, 0.38) | 0.22 (0.00, 0.43)  0.21 (0.03, 0.39) |  | 0.13 (-0.04, 0.30)  0.20 (0.05, 0.35) | 0.15 (-0.04, 0.34)  0.22 (0.06, 0.37) |
| Potassium (g / MJ) | myfood24  MPR | 0.32 (0.09, 0.54)  0.44 (0.18, 0.69) | 0.28 (0.09, 0.46)  0.35 (0.18, 0.53) |  | 0.20 (0.00, 0.40)  0.35 (0.15, 0.55) | 0.20 (0.01, 0.39)  0.33 (0.16, 0.51) |
| Sodium (g / MJ) | myfood24  MPR | 0.00 (-0.18, 0.17)  0.06 (-0.09, 0.21) | 0.00 (-0.21, 0.20)  0.07 (-0.11, 0.25) |  | 0.12 (-0.03, 0.28)  0.10 (-0.04, 0.24) | 0.13 (-0.02, 0.29)  0.11 (-0.04, 0.27) |
| Total sugars (g / MJ) | myfood24  MPR | 0.21 (0.04, 0.39)  0.30 (0.12, 0.47) | 0.26 (0.05, 0.47)  0.34 (0.17, 0.52) |  | 0.12 (-0.03, 0.27)  0.07 (-0.07, 0.21) | 0.17 (-0.04, 0.38)  0.09 (-0.09, 0.27) |

* nutrient density for protein, potassium, sodium and total sugars was expressed in grams per MJ of total energy intake

^†^ Interviewer-based multiple-pass 24-hour dietary recall

^‡^ All dietary measures and estimates were log-transformed.

Table S4. Attenuation factors and correlation between dietary assessment tool and true intake for protein, potassium, sodium and total sugar intake and density as assessed by myfood24 and interviewer-based 24-hour recall by age group.^‡^

|  | Dietary assessment tool | Age <40 years (n=87) | |  | Age 40+ years (n=125) | |
| --- | --- | --- | --- | --- | --- | --- |
|  |  | Attenuation factor  (95% CI) | Correlation with true intake  (95% CI) |  | Attenuation factor  (95% CI) | Correlation with true intake  (95% CI) |
| *Nutrient intake*: |  |  |  |  |  |  |
| Protein (g) | myfood24  MPR^†^ | 0.38 (0.25, 0.51)  0.41 (0.28, 0.54) | 0.50 (0.36, 0.65)  0.54 (0.41, 0.68) |  | 0.22 (0.11, 0.33)  0.25 (0.15, 0.36) | 0.35 (0.20, 0.51)  0.35 (0.23, 0.47) |
| Potassium (g) | myfood24  MPR | 0.33 (0.17, 0.50)  0.37 (0.20, 0.54) | 0.40 (0.22, 0.58)  0.43 (0.26, 0.60) |  | 0.27 (0.16, 0.38)  0.26 (0.14, 0.38) | 0.40 (0.25, 0.54)  0.32 (0.19, 0.46) |
| Sodium (g) | myfood24  MPR | 0.16 (0.02, 0.30)  0.19 (0.06, 0.32) | 0.23 (0.04, 0.42)  0.27 (0.10, 0.45) |  | 0.26 (0.15, 0.37)  0.16 (0.05, 0.26) | 0.36 (0.22, 0.51)  0.23 (0.08, 0.37) |
| Total sugars (g) | myfood24  MPR | 0.07 (-0.06, 0.19)  0.13 (0.01, 0.26) | 0.13 (-0.12, 0.37)  0.22 (0.02, 0.42) |  | 0.14 (0.03, 0.24)  0.17 (0.06, 0.30) | 0.23 (0.07, 0.39)  0.24 (0.11, 0.37) |
| *Energy expenditure:* |  |  |  |  |  |  |
| Total energy expenditure (MJ) | myfood24  MPR | 0.15 (0.00, 0.30)  0.24 (0.08, 0.39) | 0.22 (0.00, 0.44)  0.31 (0.12, 0.49) |  | 0.20 (0.08, 0.32)  0.27 (0.15, 0.40) | 0.31 (0.14, 0.48)  0.35 (0.21, 0.48) |
|  |  |  |  |  |  |  |
| *Nutrient density**: |  |  |  |  |  |  |
| Protein (g / MJ) | myfood24  MPR | 0.28 (0.09, 0.47)  0.33 (0.16, 0.51) | 0.32 (0.11, 0.52)  0.38 (0.20, 0.55) |  | 0.04 (-0.13, 0.21)  0.07 (-0.07, 0.21) | 0.05 (-0.14, 0.23)  0.07 (-0.07, 0.22) |
| Potassium (g / MJ) | myfood24  MPR | 0.34 (0.10, 0.58)  0.56 (0.31, 0.80) | 0.33 (0.11, 0.55)  0.49 (0.31, 0.68) |  | 0.26 (0.06, 0.46)  0.37 (0.18, 0.55) | 0.26 (0.03, 0.49)  0.35 (0.15, 0.55) |
| Sodium (g / MJ) | myfood24  MPR | -0.02 (-0.19, 0.16)  0.13 (-0.02, 0.29) | -0.02 (-0.24, 0.20)  0.16 (-0.02, 0.35) |  | 0.14 (-0.02, 0.30)  0.06 (-0.08, 0.20) | 0.15 (-0.01, 0.31)  0.06 (-0.09, 0.22) |
| Total sugars (g / MJ) | myfood24  MPR | 0.06 (-0.10, 0.22)  0.02 (-0.13, 0.17) | 0.10 (-0.17, 0.38)  0.03 (-0.21, 0.28) |  | 0.18 (0.04, 0.33)  0.25 (0.10, 0.39) | 0.23 (0.06, 0.40)  0.28 (0.14, 0.43) |

* nutrient density for protein, potassium, sodium and total sugars was expressed in grams per MJ of total energy intake

^†^ Interviewer-based multiple-pass 24-hour dietary recall

^‡^ All dietary measures and estimates were log-transformed.
